# Prediction of *Salicornia europaea* L. biomass using a computer vision system to distinguish different salt-tolerant populations

**DOI:** 10.1186/s12870-024-05743-9

**Published:** 2024-11-16

**Authors:** S. Cárdenas-Pérez, M. N. Grigore, A. Piernik

**Affiliations:** 1 Department of Geobotany and Landscape Planning, Faculty of Biological and Veterinary Sciences, Nicolaus Copernicus University in Toruń, Lwowska 1, Toruń, 87-100 Poland; 2 Doctoral School of Biology, IOSUD-UAIC, Bulevardul Carol I nr. 20A, Iasi, Romania

**Keywords:** Biomass halophyte modelling, CVS, Discriminant analysis, Image-colour segmentation, Non-destructive method, Salt-tolerance differentiation

## Abstract

**Background:**

*Salicornia europaea* L. is emerging as a versatile crop halophyte, requiring a low-cost, non-destructive method for salt tolerance classification to aid selective breeding. We propose using a computer vision system (CVS) with multivariate analysis to classify *S. europaea* based on morphometric and colour traits to predict plant biomass and the salinity in their substrate.

**Results:**

A trial and validation set of 96 and 24 plants from 2 populations confirmed the efficacy. CVS and multivariate analysis evaluated the plants by morphometric traits and CIELab colour variability. Through Pearson analysis, the strongest correlations were between biomass fresh weight (FW) *vs*. projected area (PA) (0.91) and anatomical cross-section (ACS) *vs.* shoot diameter (Sd) (0.94). The PA and FW correlation retrieved different equation fits between lower and higher salt-tolerant populations (R^2^ = 0.93 for linear and 0.90 for 2nd-degree polynomial), respectively. The higher salt-tolerant reached a maximum biomass PA at 400 mM NaCl, while the lower salt-tolerant produced less under 200 and 400 mM. A second Pearson correlation and PCA described sample variability with 80% reliability using only morphometric-colour parameters. Multivariate discriminant analysis (MDA) demonstrated that the method correctly classifies plants (90%) depending on their salinity level and tolerance, which was validated with 100% effectiveness. Through multiple linear regression, a predictive model successfully estimated biomass production by PA, and a second model predicted the salinity substrate (Sal.s.) where the plants thrive. Plants' Sd and height influenced PA prediction, while Sd and colour difference (ΔE1) influenced Sal.s. Models validation of actual *vs.* predicted values showed a R^2^ of 0.97 and 0.90 for PA, and 0.95 and 0.97 for Sal.s. for lower and higher salt-tolerant, respectively. This outcome confirms the method as a cost-effective tool for managing *S. europaea* breeding.

**Conclusions:**

The CVS effectively extracted morphological and colour features from *S. europaea* cultivated at different salinity levels, enabling classification and plant sorting through image and multivariate analysis. Biomass and salinity substrate were accurately predicted by modelling non-destructive parameters. Enhanced by AI, machine learning and smartphone technology, this method shows great potential in ecology, bio-agriculture, and industry.

**Supplementary Information:**

The online version contains supplementary material available at 10.1186/s12870-024-05743-9.

## Background

Plants experience diverse impacts from varying levels of salinity, with responses differing both between and within species. For instance, *Salicornia europaea*—an extreme halophyte—exhibits significant phenotypic variation as a plasticity strategy to adapt to different salinity environments. Despite this variability, common characteristics have been observed in populations across Northern Europe. These include an erect, intricately branched stem structure, with secondary shoots emerging from the primary cylindrical shoots, and reduced leaves, with the main assimilation area located within the shoots [1–3].

While maintaining a characteristic green hue throughout its lifecycle, the stems transform the culmination of its growth cycle, adopting a striking red hue attributed to chlorophyll reduction. This phenomenon significantly diminishes photosynthetic activity, impacting nutrient retention, biomass production, and overall hydric equilibrium [4]. However, when facing environmental salt stress, *S. europaea* experiences modifications in its physiology, morphology and anatomy. This plant employs a crucial strategy to cope with high sodium concentrations by storing water in its parenchyma cells [5]. Moreover, a reduction of growth, a thickening of shoots, and a decrease in chlorophyll pigments have been reported at higher salinities (above ~400 Mm) [2]. However, not all populations decrease these parameters at the same rate [3]. The varied salinity levels can disrupt water storage intensity, leading to significant biological effects across multiple levels. This disturbance affects various functional traits, including biomass, projected area, shoot thickness, height, foliage complexity, fractal dimension, and plant pigmentation. Of these traits, the biomass projected area obtained through image analysis was closely related to overall plant biomass development and the performance and adaptation of the plant to its environment [3, 6, 7].

Several studies [8–11] have evaluated the physiological changes that occur during salinity stress. However, the primary intention of these studies has been to find the extent of salt tolerance by evaluating the modifications in physical, anatomical and biochemical changes which require plant destructive techniques that, in most cases, also involve laborious and expensive measurements. Other studies have evidenced using chlorophyll meter instruments for plant colour measurements as a non-destructive approach [12–14]. However, these instruments have a small field of view (around 2 to 5 cm^2^), limiting the studies.

**Fig. 1:**
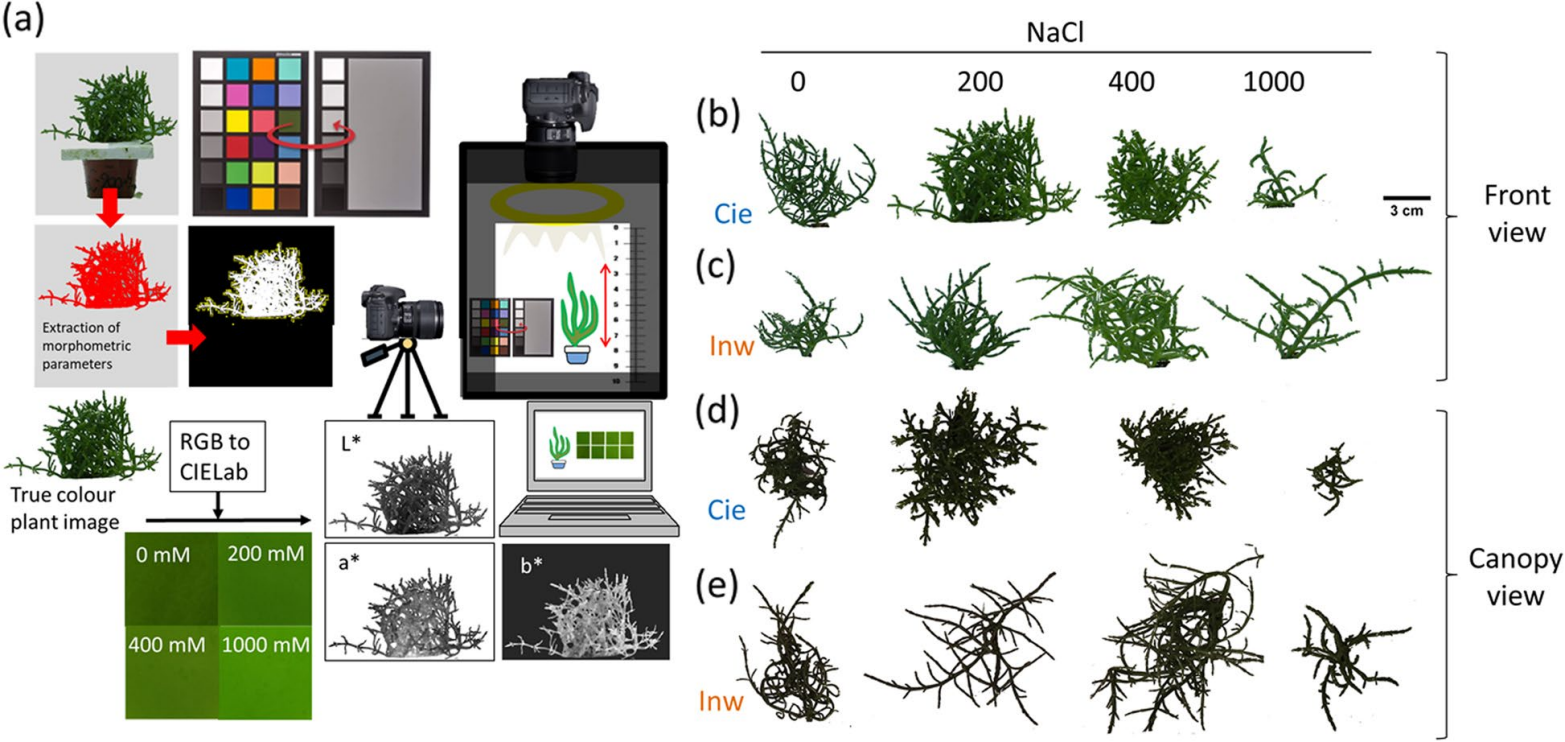
Computer vision system, image capture, processing and converting RGB to CIELab parameters (**a**). *S. europaea* plants front view images processed for image analysis for two populations Ciechocinek (**b**) and Inowrocław (**c**) cultivated under different salinity treatments (0, 200, 400 1000 mM). Canopy view, binary images from the same analysed samples, Ciechocinek (**d**) and Inowrocław (e) (*n*=12). Scale bar: 3 cm

Given the recent interest in utilising *S. europaea* as a new crop due to its diverse applications in bioenergy, food, pharmacy, and ecology sectors, alongside its numerous benefits for human consumption [15], there is a pressing need to establish a non-destructive and low-cost method for monitoring, identifying, and classifying plants based on their varying degrees of salt tolerance. Such a method would be crucial for large-scale implementation and to aid selective breeding. Non-destructive methods are emerging, especially in food technology, including near-infrared spectroscopy, hyperspectral imaging and computer vision system (CVS) [16–19]. CVS offers a rapid and cost-effective approach, exploiting conventional cameras and optimal light systems through digital image analysis (Fig. 1). This system effectively sorts samples according to the analysed traits, facilitating precise characterisation [19–21]. CVS offers several advantages over chlorophyll meters, including lower cost, potential for in-line inspection, ability to analyse larger areas for spatial data, thereby reducing human subjectivity, and the opportunity to develop automated inspection systems to also measure morphometric parameters without damaging the plant, which is helpful for industrial applications [22, 23]. Some studies have already used a CVS to inspect climacteric fruits during plant ripening and damage [24–26]. Other studies have reported similar applications on plant phenotyping using CVS with successful plant analysis and classification by using RGB standards [24, 25, 27–31]. For our purpose of colour analysis, CIELab space colour is a better choice due to its similarity to human perception of colour [32–35]

In ecology, only a few studies have been striving to discover straightforward, precise, and non-invasive techniques for assessing the impact of abiotic stressors, such as how salinity affect plants' growth functional traits [6, 7, 36–38].

Recently, scientific groups have recognised the importance of establishing advanced plant phenotyping facilities capable of automatically imaging many plants from standard positions to image analysis programs daily. Some examples are the Plant Accelerator ('Adelaide to host 5th IPPS – Australian Plant Phenomics Facility') [39], the Leibniz Institute of Plant Genetics and Crop Plant Research in Germany (IPK-PhänoSphäre - Leibniz-Institut (IPK)) [40], the PHENOPSIS system by the National Institute for Agricultural Research (INRA) in Montpellier, France ('Le système PHENOPSIS') [41].

Even *S. europaea* is becoming the new crop. To the best of our knowledge, CVS and discriminant analysis have not been employed in sorting this halophyte species based on their salinity tolerance through assessments of morphological and colour changes [2]. Herein, a study based on discriminant analysis aims to explain and predict the classification of observations into several classes using quantitative or qualitative explanatory variables. This method holds great potential for effectively classifying plants based on their salinity growth level, utilising only the most significant non-destructive features extracted from the samples. We also propose developing predictive models tailored for biomass production and the salinity content in the substrate, making plants viable environmental markers for monitoring salt lands.

To date, comprehensive studies assessing functional traits for estimating the adaptation and optimal growth of *S. europaea* along salinity gradients via non-invasive methods are lacking in the literature.

These models could be innovative and valuable tools in ecology, bio-agriculture or industrial fields with numerous applications. For instance, they can be used to improve *S. europaea* salt tolerance through selective breeding. In addition, the CVS approach can be applied to other halophytes or crops by adapting the methodology. For this case, initial experimental studies are needed to identify key functional traits under various conditions, correlating them with biochemical stress indicators. The main key for a successful system operation, lies in identifying factors affecting trait variability for each species. However, once key visible traits are identified and correlated, CVS methodology can reliably and accurately classify samples.

We hypothesised that a streamlined computer vision system will allow us to 1) discern between lower and higher salt-tolerant populations of *S. europaea* species by evaluating their salt tolerance through pivotal morphological and colour functional traits along four levels of salinity, 2) the method will cost-effectively assess and classify salt-tolerant groups through the repercussions of diverse salinity levels on functional traits, and 3) through mathematical modelling, we can predict biomass projected area (PA) and salinity in the substrate (Sal.s.) for diverse applications.

## Methods

### Plant materials, growth conditions and salt treatment

Soil samples containing *S. europaea* seeds were gathered from two distinct locations representing natural and industrial saline environments in Poland. The first site, situated in the health resort of Ciechocinek (Cie) (52°53′N,18°47′E), benefits from natural brine sources [42, 43]. The second site lies near a soda factory in the town of Inowrocław-Mątwy (Inw) (52°48′N, 18°15′E). The approximate distance between the Cie and Inw sites is 50 kilometers.

*S. europaea* seeds were collected in October 2018, and a second batch in October 2021. The seeds underwent germination in a growth chamber within Petri dishes (Ø 20 cm), each containing a piece of filter paper and 5 ml of distilled water. Following germination, they were transplanted into individual pots (height: 5.3 cm, diameter: 5.5 cm) filled with a sterile substrate blend of vermiculite and sand in a 1:1 ratio. Each pot served as an experimental unit, accommodating 12 seedlings for each salt treatment. Preceding transplantation, batches of 12 pots were arranged on trays without drainage and fully saturated with solutions containing 0, 200, 400, and 1000 mM NaCl (approximately 500 ml per 12-pot set). The pots were rotated each day to avoid positional effects. Leveraging insights from prior research, we discerned salinity tolerance levels within these populations, categorizing them as follows: 0 denotes No Salinity (NO-S), while the range of 200-400 mM NaCl represents the optimal spectrum, low and high optimum salinity (L-OS and H-OS, respectively), 1000 mM designates Severe Salinity (SS). Building upon previous findings, we categorised the Cie as lower salt-tolerance and the Inw population as higher salt-tolerance [2, 3].

The plants were cultivated in a growth chamber under controlled conditions, maintaining a day/night temperature of 25/20 °C, a photon flux density of 1000 mmol m^-2^s^-1^, relative humidity ranging between 50–60%, and a photoperiod of 16/8 h (light/dark). Seedlings received irrigation by pouring distilled water into the tray for 21 days. Subsequently, they were watered every two days with an equal volume of Hoagland's solution to ensure uniformity in both salinity and nutrient provision. A total of 96 plants constituted the trial set. Therefore, a complete randomised factorial design 2^4^ was used (4 treatments × 2 populations, 12 plants each). Following 60 days of growth, morphometric and colour parameters were assessed in 12 samples. The above-ground part of three replicates of plants per treatment were weighed to determine each population's biomass in grams of fresh weight (FW). Later, samples were oven-dried for 72 h at 85 °C, and dry weight (DW) was determined.

In a subsequent phase, an additional batch of 24 plants, the validation set, were cultivated under the same conditions as the trial set. The validation set will be used to confirm the validity of the experiments (comprising three samples per treatment for each population, totalling 4x2x3). For this set, colour and morphometric image analysis were conducted solely.

The voucher specimen of the plant material has been archived in the publicly accessible herbarium at Nicolaus Copernicus University in Toruń, identifiable by the Index Herbarium code TRN. Although the deposition number is currently unavailable, formal identification of the plant species was conducted by Dr. hab. Agnieszka Piernik, Prof. NCU. Additionally, authorization to handle the seeds was granted by the Regional Director of Environmental Protection in Bydgoszcz under the codes WPN.6205.159.2014.KLD, WPN.6205.69.2015.KLD, WPN.6205.44.2016.KLD, and WOP.6400.17.2020JC.

### Anatomy of *S. europaea* stems cells by image analysis

From the fleshy middle segment of the primary branch of *S. europaea* plant treatments (0, 200, 400, and 1000 mM NaCl) (Fig. S2), thin slices (0.5 mm) of fresh tissue were obtained using a bi-shave razor blade following the steps reported by [3, 44]. They were observed through a light microscope (LM) (Olympus BX51, USA) and a digital camera (DP72 digital microscope camera). The LM images were captured at a magnification of 10×/0.30 in RGB scale and stored in TIFF format as it allows to store more refined and detailed images, at 1280×1024 pixels. A total of 12 individuals per treatment were analysed. Image analysis (IA) was performed in FIJI ImageJ v. 1.47 software (National Institutes of Health, Bethesda, MD, USA) [45]. The anatomical cross-section (ACS) epidermis, palisade tissue, water storage parenchyma, vascular bundles with sclerenchyma and pith parenchyma were estimated as the number of pixels.

### Non-destructive approach

#### Computer vision system for capturing and analysing morphometric and colour traits from digital images

In this study, the Computer Vision System (CVS) utilized a PULUZ photography light box (model PU5060, HITSAN, China) outfitted with two 30W, 5500K integrated LED lights. These lights were designed to soften and diffuse light, effectively eliminating glare. The box's wall material acted as a diffuser, ensuring uniform illumination of the sample. Positioned in front and from a top-down perspective of the analysed plants was a Fujifilm FinePix S2000HD digital camera (13 MP, f/2.0 aperture, 1/3" sensor size, 1.12 μm pixel size, focal length of 3.79 mm) with autofocus capability (Fig. 1).

After 60 days of growth, samples (the whole plants in pots) were placed inside the CVS (Fig. 1a). The camera was located 50 cm from the samples, and front and canopy views were captured, as shown in Figure 1b-e. Consistent light and distance parameters were maintained for capturing the aerial parts of the plants across all treatments. Each treatment was replicated 12 times for both the Cie and Inw populations.

The procedure to obtain colour images and morphometric parameters using CVS consisted of three main steps: capture, processing and analysis. However, the camera must be first calibrated for colour acquisition, as reported in a previous study [46]. For this purpose, the image of a calibrated colour checker (Spyderchekr) with 24 colour patches was first captured. Then, the camera settings were calibrated following the Spyderchekr software version 1.6. The camera settings were manual mode, exposure level 0.0, ISO100 and shutter speed 1/60 without zoom or flash [26] (Fig. 1a). In this mode, the white balance was manually set to the White Fluorescent Light option. For this work, RGB images were captured with a resolution of 3648 x 2432 pixels (equating to 0.1mm/pixel) in JPEG format with the option of superfine quality and later stored in the computer in TIFF format. Previous works indicate that CIELab space is the most appropriate for measuring plants' colour changes due to environmental factors [33–35]. The CIELab space is known to represent the colours naturally as perceived by human eyes, and they are perceptually more uniform than RGB, able to detect fine changes in the plants’ pigments caused by salinity [47, 48]. Conversions from RGB to CIELab colour space were done by using the colour space converter plug-in of the ImageJ software through equations that correspond to an illuminant D65.

The CIELab coordinates include a*, representing the green-red axis, and b*, indicating the blue-yellow axis. Negative a* values indicate green, while positive values signify orange and red. Negative b* values are related to blue, and positive values to yellow. L* measures luminosity (0 = black, 100 = white) [47, 49].

The values of chroma (S*) and hue angle (Hue ) were calculated as follows:

${S}^{*}= \sqrt{({a}^{*2}+{b}^{*2})}$(eq.1) and $Hue=\text{arctan }(\frac{{b}^{*}}{{a}^{*}})$ (eq.2). Colour differences (ΔE) were calculated according to previous study [2] using the following equation: ΔE = $\sqrt{{{(\Delta L}^{*})}^{2}+{{(\Delta a}^{*})}^{2}+{{(\Delta b}^{*})}^{2}}$ (eq.3), where Δ ${L}^{*}$= ${L}^{*}$- ${L}_{0}^{*}$; Δ ${a}^{*}$= ${a}^{*}$- ${a}_{0}^{*}$;Δ ${b}^{*}$= ${b}^{*}$- ${b}_{0}^{*}$. For ΔE1, the initial colour parameters used was a white standard, while for ΔE2 the initial colour parameters correspond to values from non-salinity NO-S.

A trial of colour correlations between a colorimeter and image analysis was performed. For this purpose, a colorimeter device (PCE-XXM 30, CM700d) was used to confirm the colour calibration of our camera. We used a view angle of 10°, standard illuminant D65, and the CIELab colour space. For each of the measurements of colour, three readings were performed per sample by locating a bed of *S. europaea* aerial parts, randomly selected shoots and branches, thereby obtaining an average of the three readings (L*, a* and b*).

Trial test colorimeter values were correlated with those obtained from the image analysis and confirmed the accuracy of the colour-image analysis through a proper fit R^2^ ≥ 0.97 (Fig. S1). The images and colorimeter values were captured in triplicate per treatment for both populations.

For morphometric analysis, the same images employed for colour analysis underwent conversion to grayscale and then to binary format through manual segmentation, effectively separating the plant from the background. This segmentation process involved adjusting the threshold from 135 to 240, this threshold range was determined by selecting the frequency distribution of pixel intensities that best represents the image histogram. Then, it resulted in cropped grayscale images of the individual plants. Subsequently, plant shape and size were derived from these binary images. All image processing steps were executed using ImageJ software (Fig. 1). The biomass projected area (PA) was determined by quantifying the number of pixels enclosed within the plant's perimeter. Meanwhile, shoot diameter (Sd) was measured as the horizontal distance between the two furthest points on the middle segment of the shoot. Shoot height (Ht) represented the vertical distance from the base to the apex of the plant. Additionally, fractal dimension (FD) analysis was employed to assess the structural complexity of plant growth, a metric widely utilized in various studies to analyse the intricacies of biological samples [2, 46, 50, 51]. In the present study, FD was evaluated using the fractal box count plug-in in ImageJ [2], where higher FD values denote more complex images. Later, FW and DW vs PA (canopy and front view) parameters were plotted to ascertain if the biomass projected area (PA) serves as a non-destructive and quantifiable parameter for assessing plant biomass production.

### Statistical methods, multivariate analysis and modelling

A two-way ANOVA comparing treatments within populations and populations within treatments was conducted for all the results with the Holm–Sidak method using SigmaPlot software version 11.0 [55].

In order to seek the projection according to which the functional traits under the influence of salinity in *S. europaea* are best correlated a Pearson correlation matrix was used. For this analysis, biochemical treatments from previous research [2] conducted on the same populations were used, specifically proline (Prol), hydrogen peroxide (HP), chlorophyll a (Cha), chlorophyll b (Chb), total chlorophyll (TCh) and carotenoids (Car) against non-destructive parameters (morphometric and colour traits). In total, 18 variables were used: ten non-destructive (PA, Sd, Ht, FD, ΔE1, L*, a*, b*, Hue, S*) and eight destructive (FW, Prol, HP, Cha, Chb, TCh, Car and ACS) arranged in a matrix of the average values for the trial set: 96 plants. A second Pearson correlation matrix together with its PCA was conducted, incorporating solely the most significant non-destructive variables (PA, Sd, Ht, ΔE1, L*, a*, b*, S*, and Hue) to assess their potential for accurate salinity level classification. The relationships between variables were analysed using Principal Component Analysis (PCA). Prior to this, a significance test (KaisereMeyereOlkin) was conducted to identify variables with significant correlations (α ≤ 0.05). This involved calculating variable contributions, factorial loadings, eigenvalues, and the percentage of variance explained. Once the salinity classification was established, a supervised classification model through Multivariate Discriminant Analysis (MDA) was implemented to validate the efficacy of the developed model in sorting plants based on their salinity level by using non-destructive parameters. To evaluate the model's performance, cross-validation was conducted using 96 plants from the trial set, another group of 24 plants served as the validation set. The PCA and MDA was developed using XLSTAT software version 2023.1.4 [52]. The usefulness of the CVS with PCA and MDA methodology has been already proven in the classification of fruits in laboratory scale and in industrial lines [26, 48, 53, 54]. Predictive models were developed through multiple linear regression analysis [55]. For model development purposes and in order to create a simplified equation, the colour parameters were narrowed to ΔE1, since the colour difference parameter ΔE1 encompasses the other colour parameters—L*, a*, b*, S*, and Hue. To evaluate the generalization or estimation error of a predictive model, cross-validation was employed. This approach estimates the generalization error, denoted as L(y, ŷ), where L represents the distance function, and ŷ is the model's prediction applied to an independent test sample drawn from the distribution of x and y.

## Results

### Anatomical response comparison under four salinity levels

**Fig. 2:**
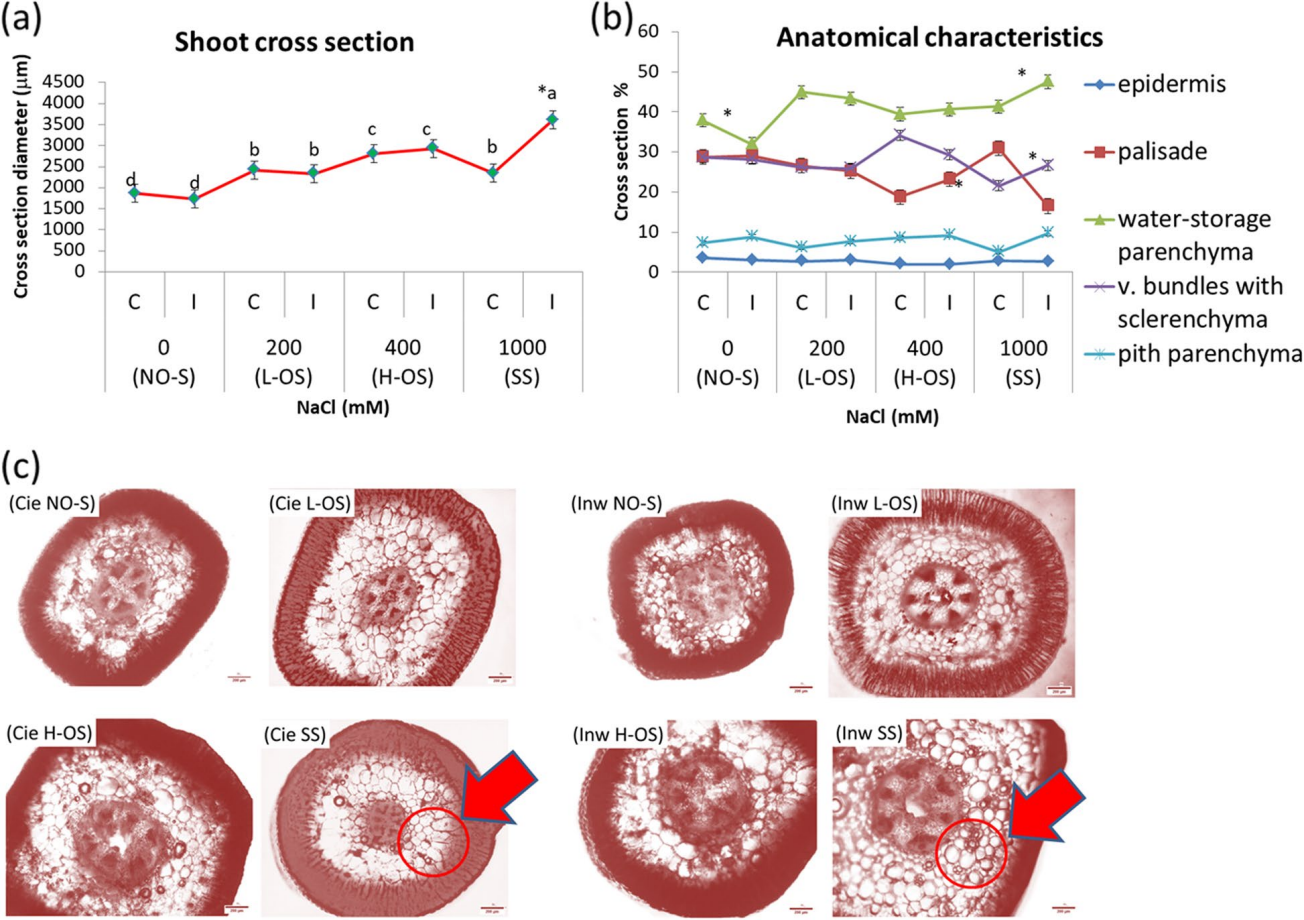
Shoot cross-section of *S. europaea* plants cultivated under four salinity treatments indicating significant differences between populations at SS (**a**), anatomical characterisation with its respective shoot cross-sections percentage for each studied parameter (**b**) (evaluating epidermis, palisade tissue, water-storage parenchyma, vascular bundles with sclerenchyma and pith parenchyma sections), salinity treatments evaluated: NO-S: no salinity, L-OS: low optimum salinity, H-OS: high optimum salinity and SS: severe salinity, for Cie and Inw populations highlighting with an arrow the marked difference at SS, Inw increased water-storage parenchyma and reduced palisade tissue, Cie increased palisade tissue (**c**). Scale bar: 200 μm. Different letters indicate differences between treatments and * between populations (*p*< 0.05). Values are the means ± SD (*n* = 12)

The anatomical traits revealed that the shoots of both populations possess a well-developed central cylinder, with a significantly higher percentage of water-parenchyma and vascular tissue containing sclerenchyma at the optimal salinity range (L-OS and H-OS). In this range, plants from both populations exhibited significantly larger shoot cross-section diameters (approximately 2500 μm) compared to those at non-optimal salinity (NO-S), which measure around 2000 μm (Fig. 2a). Within these cross-sections, there was a notable increase in water-storage tissue (Fig. 2b). However, at severe salinity (SS), the two populations responded differently. Inw showed an increase in cross-section diameter to approximately 3500 μm, with water-storage parenchyma making up 48% of the cross-section with a decreased palisade tissue 16%, Cie population had a lower cross-section diameter of ca. 2300 μm. It significantly increased the palisade tissue to 31% and reduced the vascular tissue with sclerenchyma and pith to 21%,. The water-storage parenchyma remained in 40% (Fig. 2b-c).

### CVS to evaluate morphometric growth and colour changes under four salinity levels

**Fig. 3:**
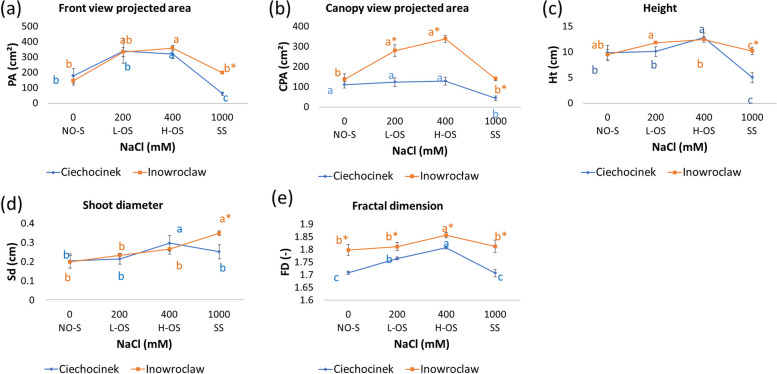
Morphological features of *S. europaea* plants cultivated under four salinity treatments, parameters obtained through CVS exhibited significant differences at SS. Front view biomass projected area (**a**), Canopy view biomass projected area (**b**), Height (**c**), Shoot diameter (**d**) and Fractal dimension (**e**). Under SS, both populations showed remarkable differences in all the analysed features. Different letters indicate differences between treatments and * between populations (*p*< 0.05). Abbreviations: PA – biomass projected area, CPA – Canopy projected area, Sd – shoot diameter, Ht – shoot height, FD – fractal dimension. Values are the means ± SD (*n* = 12)

The projected plant area was based on a front and a canopy view image (Fig. 1b-e). We did not find significant differences in front view PA, Ht and Sd between populations growing under NO-S and optimum salinity range (L-OS and H-OS). However, canopy view biomass projected area (CPA) and FD were significantly different under all the treatments for both populations (Fig. 3a-e). The plant biomass projected area (canopy and front view) was the highest for Cie and Inw in the optimum salinity range (Fig. 3a and b). Cie plants which grew under SS (1000 mM NaCl) were significantly smaller than those grown in other salinity treatments. In contrast, Inw plants subjected to the same SS treatment exhibited enhanced salt tolerance by showing intensified vigour. In SS, all morphological features significantly differed between both populations (Fig .3).

### Biomass response and correlation with projected area for lower and higher salt tolerant population

**Fig. 4:**
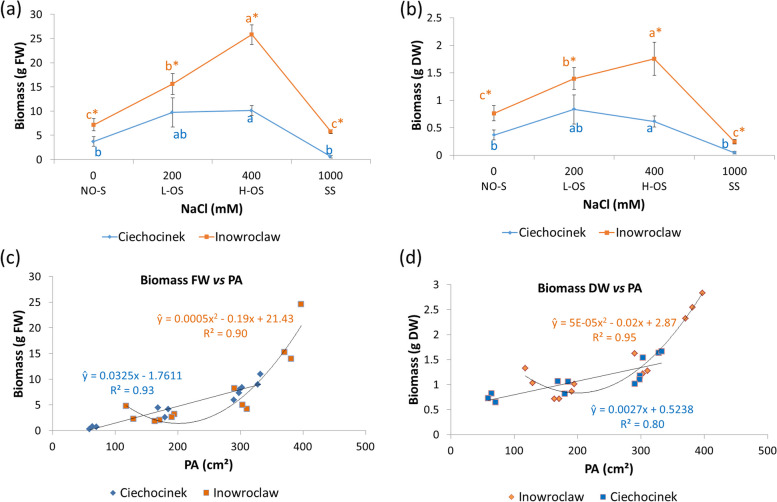
*S. europaea* biomass of plants cultivated under four salinity treatments, fresh weight FW (**a**) and dry weight DW (**b**) for Ciechocinek and Inowrocław populations. Relationship between projected area (PA) *vs* plants biomass FW (**c**) and DW (**d**) with its corresponding biomass estimation (ŷ) equation for Ciechocinek a linear fit correlation was obtained (**FW**: ŷ = 0.0325x - 1.7611, R^2^ = 0.93; **DW**: ŷ = 0.0027x + 0.5238, R^2^ = 0.80) and for Inowrocław a 2nd-degree polynomial fit (**FW**: ŷ = 0.0005x^2^ - 0.19x + 21.43, R^2^ = 0.90; **DW**: ŷ = 5E-05x^2^ - 0.02x + 2.87, R^2^ = 0.95), along the salinity gradient. Different letters indicate differences between treatments and * between populations (*p* < 0.05). Values are the means ± SD (*n* = 12)

Fresh and dry-weight biomass demonstrated different yields for each population along the four salinity treatments (Fig. 4a-b). Our analysis revealed a robust positive correlation between the projected area and biomass FW and DW across both populations. Through biomass estimation (ŷ), we derived two distinct adjustments. Lower salt tolerant has a linear correlation with a good fit R^2^ =0.93 and 0.80, for FW and DW, respectively. However, higher salt tolerant resulted in a better fit with a 2nd-degree polynomial equation R^2^= 0.90 and 0.95 for FW and DW, respectively (Fig. 4c-d). The higher salt-tolerant population (Inw) expressed its maximum PA and biomass content at 400 mM NaCl, reaching ca. 25 g FW and 1.5 g DW, while the lower salt-tolerant population (Cie) produced ca. 10 g FW and 0.7 g DW in the range of 200 and 400 mM NaCl.

### Colour analysis under salinity stress

**Fig. 5:**
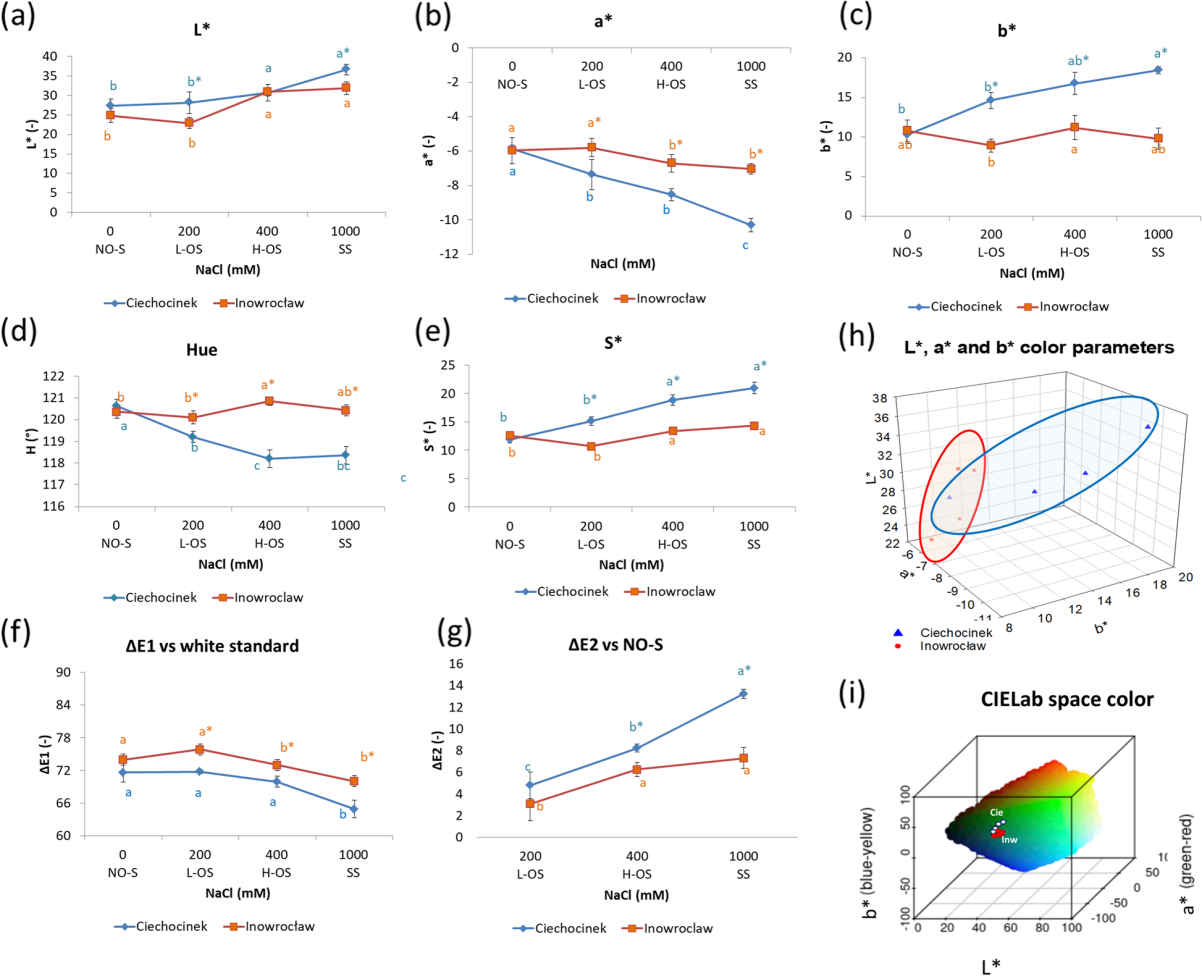
*S. europaea* colour changes of plants cultivated under four salinity treatment, all colour parameters were obtained through CVS. **a** Symbols indicate average values of L* (a), a* (**b**), b* (**c**), Hue (**d**), Chroma S* (**e**), ΔE1 the colour difference between CIELab parameters *vs* white standard (**f**) and *vs* NO-S treatment ΔE2 (**g**), 3D distribution of CIELab parameters for both populations (**h**), Sample values localisation in the CIELab space colour spectrum (**i**). Indicating that almost all the colour parameters analysed retrieve significant differences among populations along the salinity gradient, Cie showed remarkable differences between salinity treatments. Different letters indicate differences between treatments and * between populations (*p* < 0.05). Values are the means ± SD (*n* = 12). Abbreviations: ΔE1 – colour difference of L*, a* b* to white standard, L*- luminosity, a*- represents the green-red light axis, b* - indicates the blue-yellow axis, S*- chroma value/saturation, Hue – Hue angle index

The L* coordinate (Fig. 5a) measured the plant's luminosity. Starting with values L* between ca. 27 and 25 at the NO-S for Cie and Inw, respectively. L* values gradually increased until a maximum was reached on SS due to the colour change from dark green to light green. The detected change was greater in the Cie population. Significant differences were found at SS between populations. Both populations behave similarly at NO-S, but significant differences were identified at L-OS, H-OS and SS levels (Fig 5a). The values of a* decreased progressively, but more significant changes were observed for the lower salt-tolerant population compared to the higher salt-tolerant one as follows (ca. a*= -6 to -10) and (a*= -6 to -7) (Fig. 5b). On the other hand b* increases gradually, both started ca. 10.5 in NO-S, Cie reached the highest values ca. 19 at SS. Inw maintained the same value through the salinity gradient (Fig. 5c). Figures 5d-e describe the evolution of the Hue and S* values of the epidermis of the plants. Significant changes in the Hue coordinate revealed how the plant's colour is strongly influenced by salinity levels. The lower salt-tolerant population decreased from NO-S to SS with ca. 120° to ca. 118° while the higher salt-tolerant population maintained a Hue ca. 120°. The S*distinguish differences between NO-S and L-OS against H-OS and SS for both populations. Significant differences in CIELab within populations were evident throughout all the salinity gradients. All graphs indicate that the most remarkable differences between populations occur at SS. Within salinity levels, Cie displayed more significant changes than Inw, as evidenced in ΔE1 (Fig. 5f), applicable to recognise the significant differences in the whole space spectrum (L*, a* and b*). ΔE2 visualizes the variations in L*, a*, and b* between NO-S and the other salinity levels (Fig. 5g). The ΔE2 increases along with salinity, from H-OS to SS, significant differences between populations were detected. Figure 5h displays the three colour parameters in a 3D colour space, where a broader range for Cie compared to Inw is shown. Additionally, Figure 5i presents these parameters within a 3D CIELab plot, illustrating the specific region of the visible spectrum where the plants are situated and how their position shifts in response to salinity.

### Principal component analysis and multivariate discriminant analysis

In Table 1 we present the Pearson correlation matrix, highlighting in green the significant correlation coefficients observed between non-destructive and conventional biochemical parameters [2] (α ≤ 0.05). For instance, Sd notably correlated with proline (Prol) (0.75) and anatomical cross-section ACS (0.94). In contrast, PA strongly correlated with biomass FW (0.91) and inversely with hydrogen peroxide (HP) (-0.60). HP as a stress biomarker denote significant negative correlations with PA, Ht and ΔE1, a*, and positive with L*, b* and S*

**Table 1:**
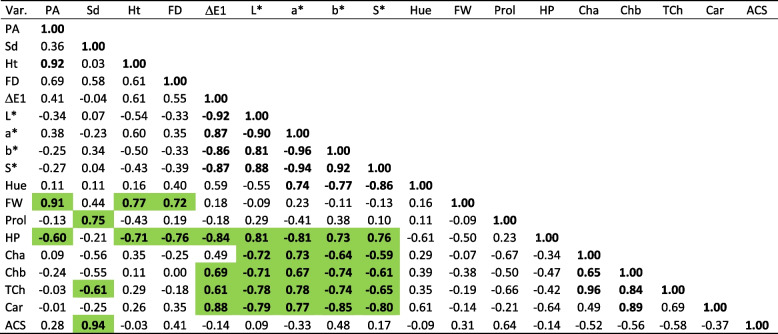
Pearson correlation matrix comparing conventional *vs.* non-destructive parameters

Highlighted in green are the most significant correlation coefficients observed between non-destructive and conventional parameters The values in bold differ from 0 with a significance level a ≤ 0.05. Abbreviations: PA – biomass projected area, Sd – shoot diameter, Ht – shoot height, FD – fractal dimension, ΔE1 – colour difference of L*, a* b* to white standard, L*- luminosity, a*- represents the green-red light axis, b* - represents the blue-yellow axis, S*- chroma value/saturation, Hue – Hue angle index, FW – above-ground fresh weight, Prol – proline, HP – hydrogen peroxide, Cha – chlorophyll a, Chb – chlorophyll b, TCh – total chlorophyll, Car - carotenoids, ACS – Anatomy Cross Section

Subsequently, a second Pearson correlation matrix was conducted (Table 2), incorporating the most significant and readily accessible parameters derived from selected non-destructive variables: PA, Sd, Ht, ΔE1, L*, a*, b*, S*, and Hue, with a focus on enabling purely non-invasive testing.

**Table 2:** Pearson correlation matrix using only non-destructive parameters

Variables	PA	Sd	Ht	ΔE1	L*	a*	b*	S*	Hue
PA	**1.00**								
Sd	-0.14	**1.00**							
Ht	**0.79**	**-0.46**	**1.00**						
ΔE1	0.28	**-0.62**	**0.64**	**1.00**					
L*	-0.30	**0.62**	**-0.61**	**-0.99**	**1.00**				
a*	0.32	**-0.58**	**0.43**	**0.66**	**-0.76**	**1.00**			
b*	-0.15	0.29	-0.16	**-0.51**	**0.64**	**-0.89**	**1.00**		
S*	-0.19	0.36	-0.22	**-0.55**	**0.68**	**-0.93**	**1.00**	**1.00**	
Hue	-0.12	0.33	-0.30	0.02	-0.12	0.27	**-0.66**	**-0.59**	**1**

The values in bold are different from 0 with a significance level α≤0.05 Abbreviations: PA – biomass projected area, Sd – shoot diameter, Ht – shoot height, ΔE1 – colour difference of L*, a* b* to white standard, L*- luminosity, a*- represents the green-red light axis, b* - indicates the blue-yellow axis, S*- chroma value/saturation, Hue – Hue angle index.

**Fig. 6:**
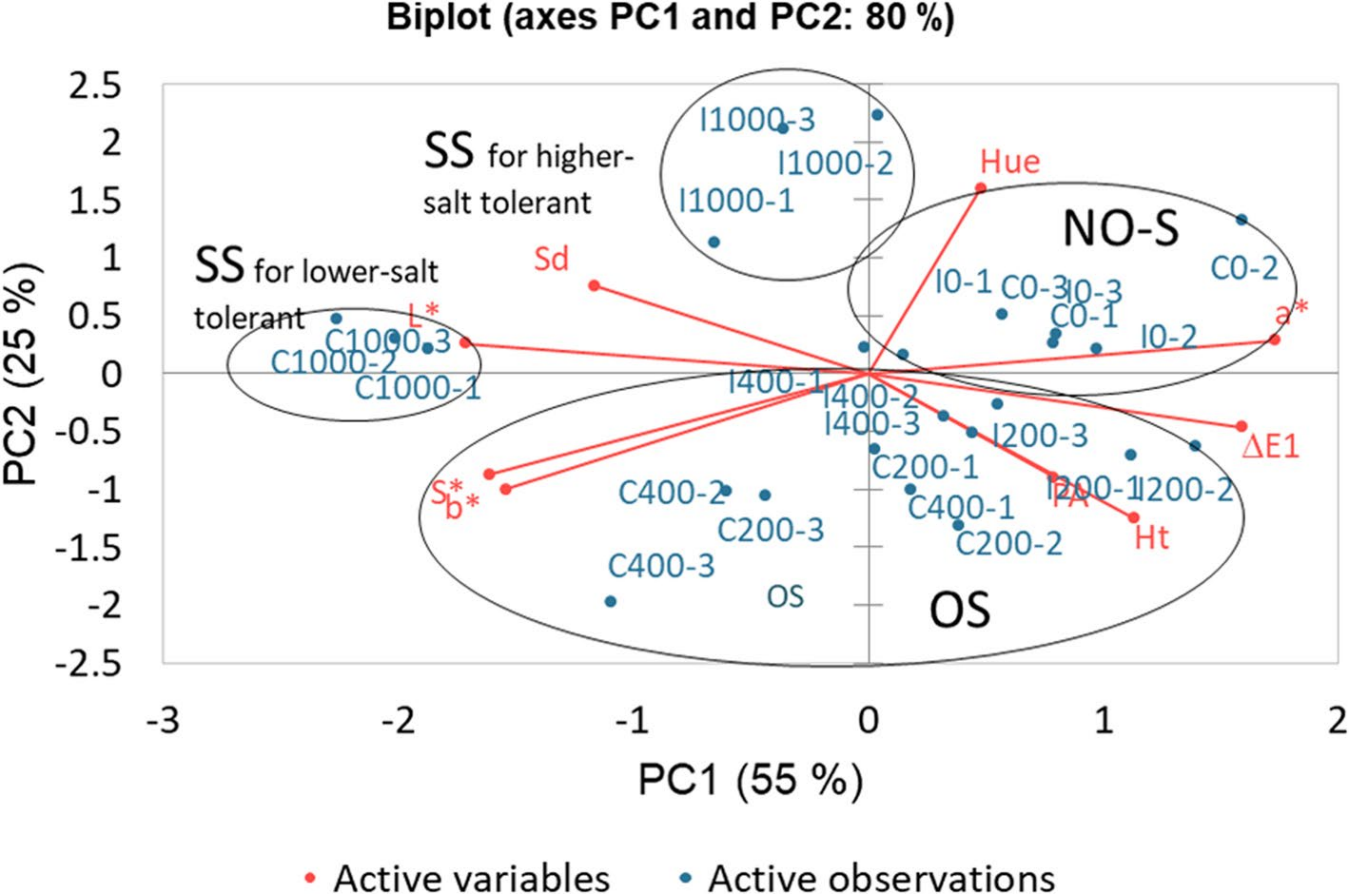
Scatter plot of the first two principal components, observations are appropriately described 80% by nine non-destructive variables, PC1 and PC2 is best represented by Sd, ΔE1, L*, a*, b*, S*, PC2 and PA, Ht and Hue respectively, properly discerning four groups: NO-S: No salinity, OS: Optimum salinity, SS: severe salinity for lower and higher salt tolerant populations. Abbreviations: PA – projected area, Sd – shoot diameter, Ht – shoot height, ΔE1 - colour difference of L*, a* b* to white standard, L*- luminosity, a*- represents the green-red light axis, b* - represents the blue-yellow axis, S*- chroma value/saturation, Hue – Hue angle index

The scatter plot of the two principal components is presented in Fig. 6. PC1 signifies the line that best represents the shape of the projected points, in this case Sd, ΔE1, L*, a*, b*, S* are the variables that better describe the characteristics of the samples in a 55%, then PC2 accounts for the next highest variance in the dataset considering PA, Ht, Hue with a 25% complementing PC1 to achieve the best description of the observations. Eigenvalues and variable contributions from morphometric and colour parameters were calculated, they showed their impact on describing the active observations for plants with lower and higher salt tolerance.

**Fig. 7:**
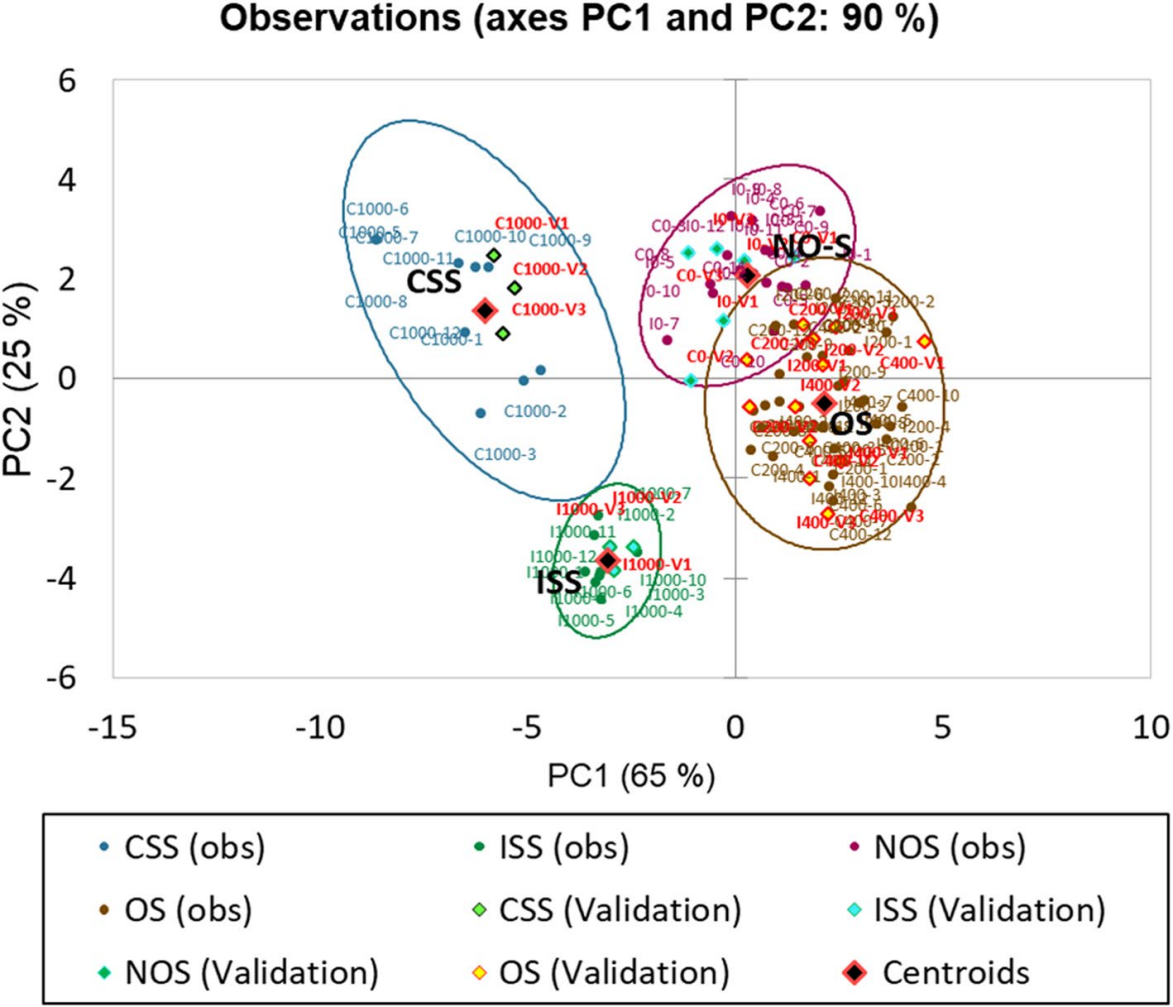
Multivariate discriminant analysis scatter plot displaying a successful identification of four *S. europaea* plant groups according to the salinity level and their tolerance with a 90% confidence interval when morphometric (PA, Sd, Ht) and colour variables (ΔE1, L*, a*, b*, S*, Hue) were applied . The salinity levels were identified as follows: NO-S: No Salinity (0mM), OS: Optimum Salinity (200-400 mM), CSS: (Severe salinity 1000 Mm in Cie, lower salt-tolerant population) ISS: (Severe salinity 1000 mM in Inw, higher salt-tolerant population). The rhombus symbol correspond to the validation set indicating a successful classification (100%) of the (a trial 96 and 24 validation set of tested plants). Abbreviations: PA – biomass projected area, Sd – shoot diameter, Ht – shoot height, ΔE1 - colour difference of L*, a* b* to white standard, L*- luminosity, a*- represents the green-red light axis, b* - represents the blue-yellow axis, S*- chroma value/saturation, Hue – Hue angle index

The multivariate discriminant analysis (MDA) represents the observations on the factor axes with a proper classification of salinity groups (Fig. 7). The PC1 is mainly described by ΔE1, L*, Ht and PA, it explains the variability of the observations in a 65%, while Sd, a*, b*, S* and Hue complement the description of the observations in PC2 with a 25%, *S. europaea* samples are discriminated in a 90%, enabling the classification of the salinity groups and population tolerance. In Table 4 the confusion matrices for the training and validation results are shown as well as the cross-validation results by comparing the correlations between the tested parameters of the plants: PA, Sd, Ht, ΔE1, L*, a*, b*, S*, Hue.

**Table 3:** Eigenvalues and factor loadings for non-destructive parameters only. In bold are those parameters with the highest variable contribution and factor loading for each principal component. A total variance of 80% is shown

	Variable contribution %				Factor loadings
	PC1	PC2		PC1	PC2
PA	3.657	**10.607**	PA	0.423	-0.484
Sd	**8.034**	7.506	Sd	**-0.627**	0.408
Ht	7.595	**20.609**	Ht	0.610	**-0.675**
ΔE1	**14.947**	2.856	ΔE1	**0.856**	-0.251
L*	**17.358**	0.900	L*	**-0.922**	0.141
a*	**17.670**	1.085	a*	**0.930**	0.155
b*	**14.059**	13.051	b*	**-0.830**	-0.537
S*	**15.320**	9.900	S*	**-0.866**	-0.468
Hue	1.361	**33.485**	Hue	0.258	**0.861**
PC Data	Eigenvalue	Eigenvalue		Variance %	Variance %
	4.90	2.21		54.45	25.59

### Model development

We obtained two multiple linear regression models based on the accurate classification of *S. europaea* groups ruled by salinity. The first model effectively predicted the biomass projected area (PA) of the halophyte influenced by salinity. The second model predicted the salinity substrate level (Sal.s.) at which a randomly selected *S. europaea* plant sample can thrive. The correlation matrix indicated that the colour parameters of the plants (ΔE1, L*, a* and b*) strongly correlated with Sd and Ht. In contrast, PA strongly correlated with Ht and is well-defined by the salinity levels. PA and Sal.s. of the plants were estimated as a linear function for a lower and higher salt-tolerant.

**Fig. 8:**
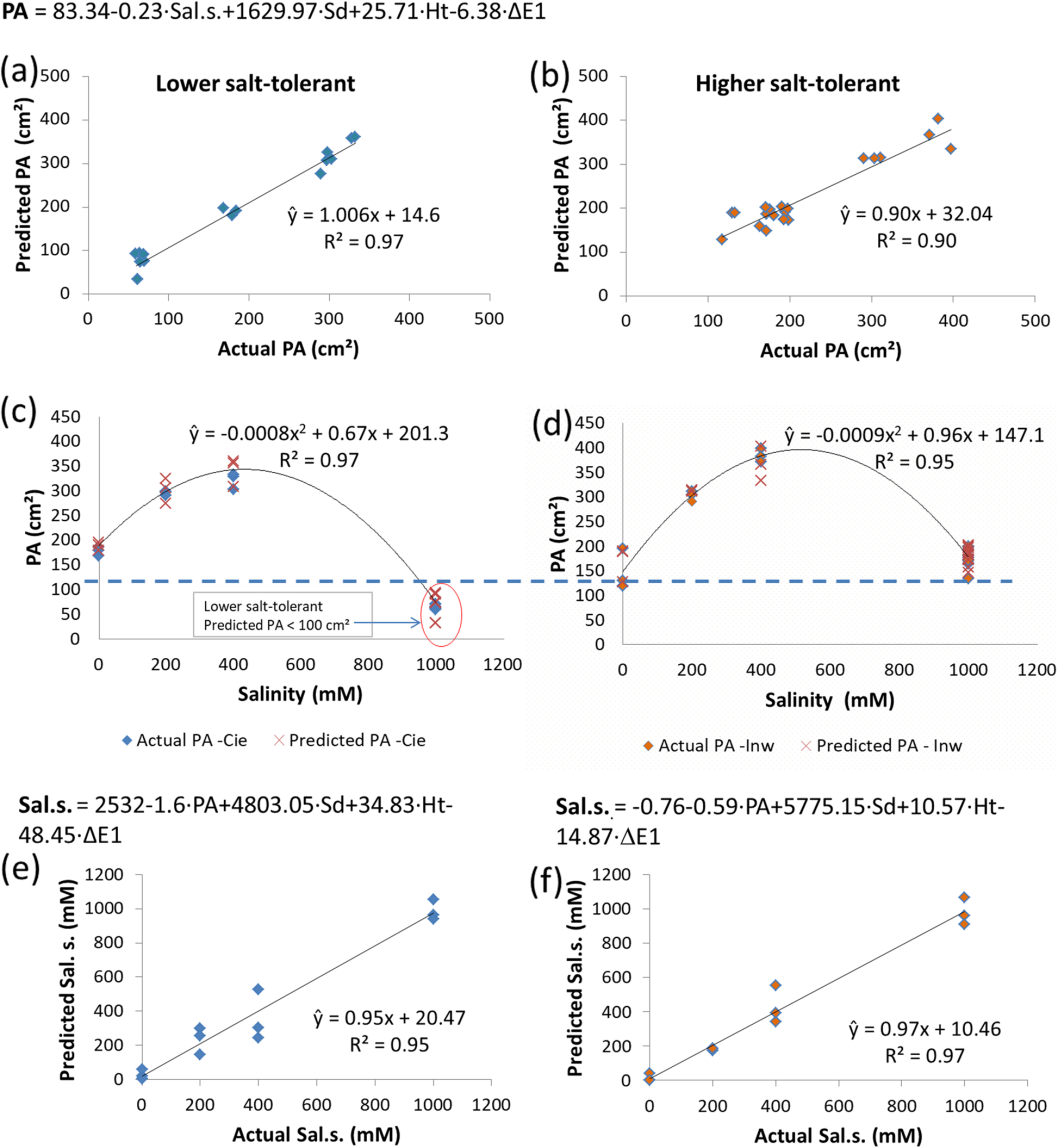
Scatter plots of actual *vs* predicted PA values of *S. europaea* cultivated under four salinity treatments for lower (**a**) and higher (**b**) salt tolerant populations, at a 5% significance level. Plots confirmed the accuracy of the performance of the predicted biomass projected area PA along the salinity gradient, compared to the actual biomass PA values for lower salt-tolerant (PA < 100 cm^2^ at SS) (**c**) and higher salt-tolerant (PA ≥ 100 cm^2^ at SS) (**d**). The equation’s fit in each scatter plot represent the estimated value "ŷ" accompanied by the corresponding coefficient of determination for all cases, values R^2^ ≥ 0.90 demonstrates an excellent model fit with the data. Scatter plots comparing actual *vs.* predicted salinity substrate levels are presented for each obtained model, showcasing the results for the lower (**e**) and higher salt-tolerant population (**f**) in both cases a R^2^ ≥ 0.95 confirmed the efficacy of the model to predict the salinity in the substrate. Abbreviations: PA – biomass projected area, Sal.s. – salinity substrate, Sd – shoot diameter, Ht – shoot height, ΔE1 – colour difference of L*, a* b* to white standard

Through multiple linear regression analysis, we obtained a general model in Eq. 4, capable of predicting PA in both populations as follows: **PA** =83.34-0.23·Sal.s.+1629.97·Sd+25.71·Ht-6.38·ΔE1 (Eq. 4) with an accurate estimation, coefficient of determination between actual *vs* predicted PA, R^2^= 0.97 and 0.90, for a lower and higher salt tolerant population, respectively (Fig. 8 a-b). We confirmed the accuracy of the actual *vs* predicted PA for each population plotted through the salinity gradient (Fig. 8c-d). Under SS conditions, the lower salt-tolerant plants exhibited a smaller PA compared to the higher salt-tolerant plants, with values dropping below 100 cm^2^ (Fig. 8c)

To forecast the salinity level at which *S. europaea* is thriving, two other predictive models were introduced in Eq. 5 and Eq. 6, one for a lower salt-tolerant and a second one for a higher salt-tolerant by using ΔE1, PA, Sd and Ht parameters (Fig. 8e-f). The coefficients of determination R^2^=0.95 and 0.97, for lower and higher salt tolerant *S. europaea* population, respectively, validated the effectiveness of using a linear regression model (Fig. 8 e-f). In Table 5 we exhibited the four selected variables correlating with PA and Sal.s. with their corresponding coefficients as probability values, highlighting those which better represents the model.

**Table 4:** Confusion matrices. Training sample matrix of 96 *S. europaea* plants retrieved a 98.96% accuracy in the classification due to similarity between NO-S and OS. The validation sample matrix of 24 plants achieved a perfect 100% classification accuracy. Cross-validation (96 x 24) successfully identified plant groups (100%) in their respective salinity level and tolerance

Training sample						
from \ to	CSS	ISS	NOS	OS	Total	% correct
CSS	12	0	0	0	12	100.00%
ISS	0	12	0	0	12	100.00%
NOS	0	0	24	0	24	100.00%
OS	0	0	1	47	48	97.92%
Total	12	12	25	47	96	**98.96%**
Validation sample						
CSS	3	0	0	0	3	100.00%
ISS	0	3	0	0	3	100.00%
NOS	0	0	6	0	6	100.00%
OS	0	0	0	12	12	100.00%
Total	3	3	6	12	24	**100.00%**
Cross-validation results						
CSS	12	0	0	0	12	100.00%
ISS	0	12	0	0	12	100.00%
NOS	0	0	24	0	24	100.00%
OS	0	0	0	48	48	100.00%
Total	12	12	24	48	96	**100.00%**

Salinity in substrate for lower salt tol. = 2532-1.6·PA+4803.05·Sd+34.83·Ht-48.45·ΔE1 (Eq. 5);

Salinity in substrate for higher salt tol.= -0.76-0.59·PA+5775.15·Sd+10.57·Ht-14.87·ΔE1 (Eq.6)

## 4 Discussion

The first novelty of this work is the use of a computer vision system as a non-destructive way to assess different functional trait responses to varying salinity gradients across populations. Halophytes like *S. europaea* are plants adapted to high-salinity soils, however depending on their environment and salt exposure, different growth responses can be achieved [3, 5, 56, 57]. Therefore an easy identification of their adaptive functional traits is crucial for swiftly understanding *S. europaea* dynamics in natural salt marsh ecosystems and for developing effective strategies to tackle salinity challenges in future agricultural practices for arid regions. [58].

According to previous research [2], conventional-invasive methods demonstrate decreased morphometric growth parameters and photosynthetic pigments with increasing salinity concentrations for two different populations. Photosynthetic pigments are traits easily disturbed by salinity during plant development, and they are considered conventional markers for evaluating plant salt tolerance between populations [59]. Notably, in our study the thickening of shoots observed through image analysis in Inw population can be attributed to the accumulation of osmoregulatory compounds, such as sugars and free proline (Figure 1 and 2) [60]. The osmoregulatory mechanism is essential for preventing water loss and mitigating ion toxicity [61, 62]. By reducing water potential, osmoregulation allows these organisms to absorb more water from their environment [5, 59, 61, 63, 64]. Our previous findings also demonstrate that the Inw population has superior coping mechanisms against hydrogen peroxide (HP) overproduction, displaying no significant differences across the initial three salinity levels, as an indicative of a reduced cellular stress.

Anatomical trait analysis also revealed significant internal differences between the two tested populations under SS. The Inw population demonstrated superior performance under higher salinity, particularly through increased water storage parenchyma, associated with the higher accumulation of proline (Figure 2). The water storage in tissue, anatomically observed, is an effective strategy for coping with severe salinity (SS). Through higher water retention as a functional trait, the plant maintain a balance between ionic uptake and water content [3, 5, 65]. The greater water storage in higher salt-tolerant indicates a trend of augmented cell size in water-absorbing tissues under moderately saline conditions [5, 66]. In this regard, the reduction of water storage parenchyma in the Cie population at SS can be explained by its reduced capacity of osmoregulation [3, 65].

Traditional methods for examining salt stress effects on plants are often time-consuming, costly, and labour-intensive. The biochemical methods can be invasive, making it difficult to perform repeated measurements on individual plants over time. This limitation underscores the need for alternative, non-invasive methods that allow for continuous monitoring and assessment.

Our non-destructive approach using CVS effectively proved that two populations of *S. europaea*, with differing levels of salinity tolerance, can be accurately classified. The sorting of plant’s salinity-tolerance was achieved by analysing the most pertinent functional traits characterising them. For instance, CVS was sensible in identifying pigment differences between populations and within treatments without damaging the plants (Figure 3), as shown in other studies where colour differences in fruits or *S. europaea* plants were analysed [2, 26, 48].

Evaluation of growth responses, as indicated by morphological properties through non-destructive methods, revealed that severe salinity SS negatively impacted these traits. Previous research reports a growth stimulation of *S. europaea* and other halophytes under optimum salinity from 200 to 400 mM NaCl [11, 67, 68]. However, when comparing between populations, significant differences were noted. The plants identified as being higher salt tolerant population (Inw) [2], retrieved higher morphological values compared to the lower salt tolerant group (Cie), as shown in the present study through front and canopy biomass projected area.

Similarly to our results, research by [11] prove that *S. europaea* growth traits are reduced when the plant grows without salinity. They also demonstrate a more significant limitation of shoot diameter and plant height at ca. 700 mM NaCl compared to lower salinity. However, in our present study, two different outcomes were obtained under severe salinity SS. The lower salt tolerant population retrieved lower height and biomass projected area than the NO-S treatment. While the higher salt tolerant population retrieved more complex front biomass projected area, height and shoot diameter than NO-S.

Our proposed method for analysing morphometric parameters efficiently distinguished salinity tolerance by recognizing the lower and higher salt-tolerant population, demonstrating high sensitivity in detecting and classifying plants across salinity levels ranging from 0 to 1000 mM, thereby confirming our initial two hypotheses.

Regarding biomass estimation, we found that by including both canopy and front biomass area perspectives during image capture, a more accurate and precise estimation of plant biomass was achieved (Figure 4). Through both views, we are considering the maximum area of plant growth influenced by sunlight orientation. Other studies have confirmed that canopy area significantly influences the accuracy of biomass estimation [57, 58]. This approach should be adopted in future biomass estimation studies when utilizing image colour analysis and modelling in plants.

The biomass PA displayed a good fit equation when correlated with conventional parameters of biomass response (fresh and dry weight). However, the two different responses, a linear *vs.* the polynomial equations variance may rely upon the distinct salt-adaptation strategies along the salinity gradient as already stated [3]. The higher salt-tolerant population reached a double FW and DW biomass production than the lower salt-tolerant (Figure 4).

The correlation between FW biomass and the projected area may still be strong throughout its development until older plants are achieved. However, further studies are needed to confirm these findings through long-term experiments and by analysing the features of plants growing directly in the field. These studies should take into account the influence of additional environmental variables, for example temperature and humidity. Our present analysis proved cost-effectiveness for assessing large-volume biomass production, whether for cultivation or for industrial purposes, without requiring sample destruction. Also, it is worth emphasising that DW is a measurement that requires oven-dried samples. Nevertheless, calculating the DW biomass of many plants using this method is time-consuming, costly, and labour-intensive. Therefore, the proposed CVS and imaging method is very suitable for inferring plant biomass FW and DW as a fast, non-destructive alternative.

Previous studies prove the utility of using biomass PA through image-based phenotyping as a non-destructive screening to identify different salinity tolerance and predict biomass production in rice [6] and cereal plants [7]. However, few studies report biomass estimation in halophytes or similar plants, for instance, a multi-scale biomass estimation of an alpine desert shrub based on relative cover and a biomass carbon estimation in halophytes [53, 69], both studies employ modelling methods and their results reveal that the models could be used to accurately represent the synergistic relationship between plant morphological variables and biomass increase at different scales. However, these approaches does not include a CVS or any other vision system as a versatile tool to efficient the process. Our study is the first to implement CVS for estimating biomass production in halophytes. Additionally, the precision of this approach can be scalable.

The colour analysis complemented and corroborated the salinity effects in photosynthetic pigments. The proposed CVS was highly sensitive in detecting colour changes between salt treatments, even when these changes were not apparent to the human eye. Across the four salinity levels, a significant shift in colour was observed, ranging from dark green to light green (Figure 5).

Regarding L*, its gradual rise was induced by salinity, resulting in the de-greening of the plants. The Cie population has higher L* at SS, meaning less chlorophyll pigments than Inw at SS. While this alteration can be visually noted as a lighter green Hue, distinguishing between populations may not be straightforward. The most remarkable changes in a* parameter (that goes from green to red) were produced for Cie at SS. L* increment and a* decrement in salinity gradient are associated with the degradation of chlorophyll pigments. On the other hand, b* that goes through +b* yellow direction, increases more intensively in the lower salt-tolerant population. According to studies related to colour change, b* values are linked to elevated levels of xanthophylls and a reduction in chlorophyll content within the chloroplasts [70]. Hue and S* parameters were functional to complement the colour screening and to identify differences within salt treatments in each population. The Hue parameter is sensitive to changes in the tone of *S. europaea* plants (in this degree range, green corresponds to ~120° and yellow to ~90°). The evolution in S* parameter evidenced the continuous and positive shift in dark green to a more saturated and uniform light green/yellow from no salinity to severe salinity. If the analysed colour has a high S*, means that virtually it does not have any hint of dark, white or grey to alter its shade, tint or tone [71].

ΔE1 results proved to be a valuable parameter for comparing the average distance of the three colour components. ΔE2 properly categorised differences within salinity treatments considering NO-S as the standard point of comparison. ΔE values below 5 indicate that the colour differences are imperceptible to the human eye [71]. Therefore, it is important to emphasizes the use of computer vision systems for quantitatively assessing delicate phenotypic changes.

The colour changes captured through CVS digital imaging reveal stress-related processes in plants non-destructively, which are difficult for a human inspector to detect. CVS colour analysis can be applied in ecology to identify weak populations under stress due to high salinity in the environment or for breeding purposes, selecting plants that are best adapted to salinity for reclaiming arid lands. Technically, it could be used to determine which plant groups are most suitable for specific processing purposes, such as biomass production, phytochemical components extraction, bioethanol and oil production. While focused on *S. europaea*, this method could be adapted for other species or crops. Combining unsupervised and supervised techniques could also identify progressive stages of stress response from image series, reducing costs and time.

Based on the findings derived from both conventional and non-destructive parameters, two distinct salt-tolerant populations were confirmed. The Pearson correlation matrix (Table 1) indicated the most significant correlations between non-destructive and conventional parameters that accurately indicate the salt-stress levels.

The subsequent Pearson correlation matrix using non-destructive parameters (Table 2) revealed the efficacy of utilising non-destructive variables to characterise salinity effects. The variable contribution and factor loadings (Table 3) served to identify those parameters that best described the *S. europaea* plant samples observations with a good score of percentage variation considered statistically reliable [72]. In the PCA scatter plot PC1 signifies the line that best represents the shape of the projected points that better describe the characteristics of *S. europaea* samples, while PC2 accounts to achieve the best description of the observations in the salinity gradient (Figure 6).

MDA validated that *S. europaea* populations are effectively discriminated along the factor axes derived from the original explanatory variables. The system properly separated the three salinity levels including two distinct groups for lower and higher salt-tolerant populations.

The MDA as a statistic tool has already been reported in a few works [26, 48, 73], those studies use a discriminant analysis to classify crops by non-destructive parameters and by scald severity based on metabolites accumulated (Figure 7). The stepwise model reported in a similar study using CVS [73] indicates that the studied crop attributes contributed significantly to separating five scald severity indexes with a confidence interval of 96.83%. While another study [26] demonstrates a 100% fruit classification from a group of 114 samples by using only colour parameters as a non-destructive method, they could sort out the ripening stages of apples.

The confusion matrix (Table 4) summarised the classification of the *S. europaea* plant observations and provided immediate insight that almost all plant samples were properly classified. We detected that only a single plant sample under OS was reclassified as NO-S. There were several ways in which these results can be interpreted when working with CVS: either the person who made the measures made an error when recording the values, or the corresponding parameter descriptors of some samples NO-S are pretty similar to samples under OS. For this study, the last assumption is more evident, so a precise separation between groups NO-S and OS was not reached. The confusion matrix for the validation group and the cross-validation group, confirmed the successful classification for both, trial and validation sets.

Through predictive modelling, biomass PA and salinity substrate (Sal.s.) can be estimated as linear functions for both low and high salt-tolerant plants (Table 5). The most significant variables for both models matched only in Sd parameter. For instance, the most weighty variables in the Sal.s. predictive model were Sd and ΔE1. We demonstrated the precision of the first model by testing actual *vs*. predicted PA with high coefficients of determination for lower and higher salt tolerant *S. europaea* populations. The coefficients indicate that a linear regression model adequately predicts the biomass PA by using only four explanatory variables: Sal.s., Sd, Ht, and ΔE1.

**Table 5:** Model parameters derived from multiple linear regression to predict *S. europaea* biomass PA and Sal.s. Model 1 predicts PA, it is best represented by Sal.s., Sd and Ht parameters (*p* **=0.00**1**,** *p<0.0001, p<0.0001*** )**. Model 2 predicts Sal.s., it is best represented by Sd and ΔE1 (*p* <0.0001, *p* =0.02). Abbreviations: Sal.s. – salinity in the substrate, PA – biomass projected area, Sd – shoot diameter, Ht – shoot height, ΔE1 – colour difference of L*, a* b* to white standard

**Model 1**	Both populations		**Model 2**	Lower salt-tolerant		Higher salt-tolerant	
Parameters			Parameters				
**PA prediction**	Coefficient value	*p* > |t|	**Salinity in substrate prediction**	Coefficient Value	*p* > |t|	Coefficient Value	*p* > |t|
Constant	83.33	0.77	Constant	2532	0.05	-0.76	0.99
Sal.s.	-0.23	**0.001**	PA	-1.6	0.15	-0.59	0.19
Sd	1629.96	**<0.0001**	Sd	4803.05	**0.001**	5775.15	**<0.0001**
Ht	25.71	**<0.0001**	Ht	34.83	0.41	10.57	0.56
ΔE1	-6.38	0.111	ΔE1	-48.45	**0.02**	-14.87	**0.02**

The relationship between observed and predicted biomass PA through the salinity levels served as compelling evidence for the accuracy of our estimation (Figure 7). Notably, a plant biomass area below 100 cm^2^ under severe salinity (SS) indicated that the plants are indeed experiencing major salinity stress. The acquired lesser plant area, suggests a population with lower salt tolerance. The proposed models show great potential for using *S. europaea* in breeding applications, enabling the differentiation of populations with varying levels of tolerance to severe saline stress. Such insights may help developers forecast suitable plant growth environments and estimate the potential biomass yield under different growing conditions.

The models also serve as a valuable ecological markers. Through estimating the physiological adaptation of plants, we are able to discern whether they are thriving under optimal conditions or facing major salinity stress in their natural habitat. The ability to gauge plant response aids ecologists in monitoring crucial soil dynamics and detecting noteworthy changes in ecological systems.

The Sal.s. model proposed in this study correctly forecast the salinity level at which the plants thrive. The respective actual *vs*. predicted Sal.s. coefficients of determination for a lower and higher salt-tolerant can be explained using only image-colour parameters. The model holds significant utility, particularly in utilising this halophyte as an environmental stress indicator.

In real-world scenarios, we can follow the steps outlined in this study, but additional dynamic calibrations may be necessary due to highly variable data. Plant responses to different photoperiod, stage development, temperature, humidity and other environmental factors. To overcome these challenges, periodic model updates will be needed to maintain accuracy. These can be addressed by augmenting training data, using transfer learning, robust discriminant analysis, and neural networks, implementing dynamic calibrations, and conducting extensive testing with simulation tools. Because, when working with robust data, it's essential to enhance the training process by simulating diverse environmental conditions.

In future studies using *S.europaea* long-term growth, a similar experiment could be developed to find the respective correlation between biomass and the projected area throughout the different growth stages until older plants are established. To address the need for long-term data, we estimate that one year will be required to monitor the plant's full developmental stages, followed by a second year dedicated to validate the accuracy of the method.

## Conclusions

The present study confirms the efficacy and advantages of using a Computer Vision System (CVS) to non-destructively acquire functional traits of *S. europaea*, including morphometric and colour parameters. The parameters extracted from CVS effectively served as functional input data for multivariate analysis, allowing for a thorough characterization and classification of the plants across different salinity levels. The proposed method accurately classified plants under low, optimal, and severe salinity, and identified a lower and higher salt-tolerant population based on their growth and colour trait responses to varying salinity levels.

The innovation of our work lies in the development of a straightforward, cost-effective, and non-destructive approach for categorizing *S. europaea*. Our research introduces accurate and practical mathematical models capable of predicting *S. europaea* biomass projected area and the salinity of the soil substrate. The study addresses the lack of image and colour analysis methods for plant stress classification by introducing a computer vision system as an innovative approach that leverages these traits for precise classification.

## Supplementary Information

Supplementary Material 1.

## Data Availability

The datasets used and/or analysed during the current study are available from the corresponding author on reasonable request.
