# Supplementary material for: Prediction of Salicornia europaea L. biomass using a computer vision system to distinguish different salt-tolerant populations
Source: BMC Plant Biol. 2024 Nov 16;24:1086. doi: 10.1186/s12870-024-05743-9 (PMC11568609; doi:10.1186/s12870-024-05743-9)
Supplement: Supplementary file 1 — Supplementary Material 1. [file 12870_2024_5743_MOESM1_ESM.pdf]

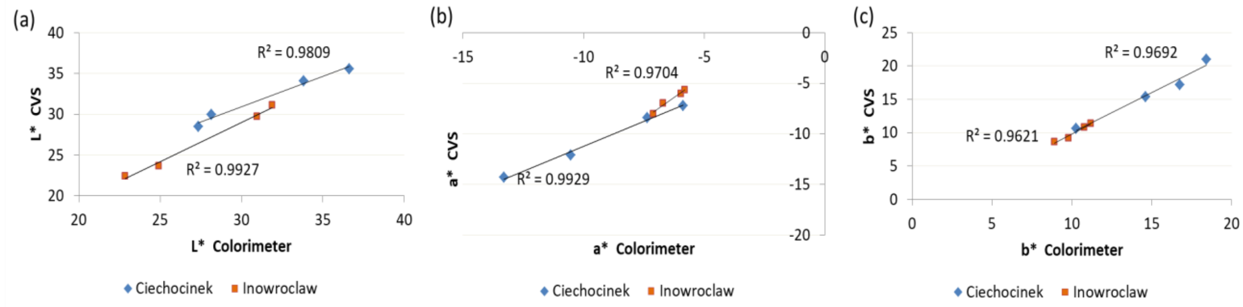


Fig.S1. Correlation of colour parameters L*(a), a*(b) and b* (c) demonstrate a proper fit $R^2 \geq 0.97$, obtained through Colorimeter *vs* CVS for the two analysed populations. *Abbreviations: L*- luminosity, a*- represents the green-red and b* represents the blue-yellow axis*
